# Evaluation of NTRK expression and fusions in a large cohort of early-stage lung cancer

**DOI:** 10.1007/s10238-023-01273-0

**Published:** 2024-01-19

**Authors:** Anne Pernille Harlem Dyrbekk, Abdirashid Ali Warsame, Pål Suhrke, Marianne Odnakk Ludahl, Nermin Zecic, Joakim Oliu Moe, Marius Lund-Iversen, Odd Terje Brustugun

**Affiliations:** 1 University of Oslo, NO-0316 Oslo, Norway; 2 Department of Pathology, Vestfold Hospital Trust, NO-3103 Tønsberg, Norway; 3 Department of Cancer Genetics, Institute for Cancer Research, The Norwegian Radium Hospital, NO-0310 Oslo, Norway; 4 Department of Pathology, Oslo University Hospital, The Norwegian Radium Hospital, NO-0310 Oslo, Norway; 5 Department of Microbiology/Division for Gene-Technology, Vestfold Hospital Trust, NO-3103 Tønsberg, Norway; 6 Department of Internal Medicine, Vestfold Hospital Trust, NO-3103 Tønsberg, Norway; 7 Department of Oncology, Vestre Viken Hospital Trust, NO-3004 Drammen, Norway

**Keywords:** Lung cancer, NTRK, Immunohistochemistry, Fluorescence in situ hybridization, Next-generation sequencing, Molecular pathology

## Abstract

Tropomyosin receptor kinases (TRK) are attractive targets for cancer therapy. As TRK-inhibitors are approved for all solid cancers with detectable fusions involving the Neurotrophic tyrosine receptor kinase (*NTRK*)-genes, there has been an increased interest in optimizing testing regimes. In this project, we wanted to find the prevalence of *NTRK* fusions in a cohort of various histopathological types of early-stage lung cancer in Norway and to investigate the association between TRK protein expression and specific histopathological types, including their molecular and epidemiological characteristics. We used immunohistochemistry (IHC) as a screening tool for TRK expression, and next-generation sequencing (NGS) and fluorescence in situ hybridization (FISH) as confirmatory tests for underlying *NTRK-*fusion. Among 940 cases, 43 (4.6%) had positive TRK IHC, but in none of these could a *NTRK* fusion be confirmed by NGS or FISH. IHC-positive cases showed various staining intensities and patterns including cytoplasmatic or nuclear staining. IHC-positivity was more common in squamous cell carcinoma (LUSC) (10.3%) and adenoid cystic carcinoma (40.0%), where the majority showed heterogeneous staining intensity. In comparison, only 1.1% of the adenocarcinomas were positive. IHC-positivity was also more common in men, but this association could be explained by the dominance of LUSC in TRK IHC-positive cases. Protein expression was not associated with differences in time to relapse or overall survival. Our study indicates that *NTRK* fusion is rare in early-stage lung cancer. Due to the high level of false positive cases with IHC, Pan-TRK IHC is less suited as a screening tool for *NTRK*-fusions in LUSC and adenoid cystic carcinoma.

**Supplementary Information:**

The online version contains supplementary material available at 10.1007/s10238-023-01273-0.

## Introduction

Biomarker driven targeted therapy has become increasingly important in cancer treatment. For lung cancer and especially for non-small cell lung carcinoma (NSCLC), it has represented a paradigm shift and has provided significant improvements in overall survival [1, 2]. Lately, targeted therapy has become relevant also for early-stage lung cancer [3]. One such target is the Tropomyosin receptor kinase (TRK). Neurotrophic tyrosine receptor kinase (*NTRK)* 1–3 are three genes that all code for a transmembrane receptor tyrosine kinase: TRK-A, TRK-B and TRK-C. *NTRK* gene fusions involving the kinase domain of the TRK protein can lead to constitutive activation of the kinase, thereby activating several signal transduction pathways that are important in carcinogenesis [4–7].

TRK-inhibitors were the first drugs to get tumor-agnostic approval by The European Medicines Agency (EMA) [8], meaning that the drug is approved for all tumors with *NTRK*-fusion, irrespective of the origin of the tumor or histopathological type. As tumor-agnostic approval results in higher testing volumes, it has led to increased interest in optimizing testing regimes for *NTRK*-fusion. Fluorescence in situ hybridization (FISH) has been regarded as the gold standard in fusion detection, but in the case of *NTRK*-fusions, a FISH-analysis will require three different probes (*NTRK*1-3). Thus, more feasible and less time-consuming methods have been sought. Immunohistochemistry (IHC) is a widely available and relatively inexpensive method [9]. Various TRK IHC clones exist. Recently, there has been particular interest in the pan-TRK IHC clone EPR17341. This antibody binds to an epitope in the kinase domain preserved in all three TRK proteins [10], thus covering protein expression from all three *NTRK*-genes in one test. Studies on this clone have found it to have a high sensitivity, particularly for *NTRK*1 and  − 2 fusions, and a specificity between 81.1 and 100% [11–14]. European Society for Medical Oncology (ESMO) recommends different testing regimes depending on the assumed prevalence of *NTRK*-fusions in the tumor type of interest [15]. In tumor types with a high prevalence of *NTRK*-fusions, ESMO recommends FISH, Real-Time Polymerase Chain Reaction (RT-PCR) or RNA-based sequencing. Where* NTRK*-fusions are less common, it recommends using IHC as a screening test with RNA-sequencing as a confirmatory test, alternatively employing RNA-sequencing as a first line test with IHC as a confirmatory test of protein expression.

In lung cancer, the prevalence of *NTRK*-fusions is considered to be low, with a prevalence between 0.16 and 0.23% [12, 16–19]. However, previous studies have mainly contained sequencing results from patients with late-stage disease and lung adenocarcinoma (LUAD). Furthermore, they have largely been based on study populations in referral institutions, which might be enriched with samples that are negative for driver mutations such as *EGFR, BRAF* or *ALK*-fusions. Thus, less is known about the prevalence of *NTRK*-fusions in early-stage lung cancer and other histopathological types beyond LUAD.

In this study, we wanted to investigate the prevalence of *NTRK*-fusion in a large cohort with early-stage lung cancer of various histopathological types. We used pan-TRK IHC as a screening tool for TRK expression, and RNA-sequencing and FISH as confirmatory tests for underlying *NTRK-*fusions. We also wanted to ascertain if the TRK IHC-positivity was associated with specific histopathological types or with certain molecular or epidemiological characteristics.

## Methods

### Patient and specimen characteristics

The study population consisted of patients with surgically resectable lung cancer admitted to surgery at Oslo University Hospital from 2006 to 2018. All the patients in the study gave written informed consent, and the Regional ethics committee approved the project (1904/2009). Biobank material was collected and clinical, molecular and histopathological information was registered prospectively.

We used whole sections from the original formalin-fixed paraffin-embedded (FFPE) blocks, tissue microarrays (TMAs) obtained from the FFPE blocks, and DNA and RNA extracted from fresh frozen material or the FFPE blocks. See Online Resource for more details about patient and specimen characteristics.

### Assay methods-immunohistochemistry

We used the VENTANA pan-TRK (EPR17341) assay (Roche Diagnostics, 790-7026). This clone detects wildtype and chimeric fusion TRK-A, B and C proteins [20]. The TMAs and the whole sections were stained at the Department of Pathology at Vestfold Hospital Trust on a VENTANA BenchMark ULTRA system. A specimen from the cerebellum was used as a positive control [20], as well as a salivary gland secretory carcinoma (ETV6-NTRK3).

We used the semi-quantitative scoring system H-score [21], which is calculated as:

(1x (percentage of relevant cells with 1 + staining) + (2x (percentage of relevant cells with 2 + staining) + 3x (percentage of relevant cells with 3 + staining) [22, 23].

In addition, we used a qualitative categorization: I: Negative II: Weak and focal III: Weak and widespread IV: Moderate/strong and focal V: Moderate/strong and widespread VI: Too few viable tumor cells (< 10 viable tumor cells) VII: Ambiguous. Weak equalled 1 + staining, moderate equals 2 + staining, and strong equalled 3 + staining. Focal and widespread described the IHC distribution pattern, where focal was defined as staining in < 50% of tumor cells and widespread is defined as staining in > 50% of tumor cells.

All the TMAs were independently examined twice by a pathologist (APHD). A random sample of five TMA blocks was reexamined by another pathologist (PS). Two pathologists (PS and APHD) analyzed all ambiguous samples and discussed them in a consensus meeting.

We defined the positive IHC as samples with weak and widespread (III), moderate/strong and focal (IV) or moderate/strong and widespread (V) staining. Negative IHC was defined as samples with totally negative (I) or weak and focal (II) staining [24].

IHC-positive samples were also examined with whole section IHC to look for heterogeneity in staining. Whole section IHC was also conducted on a subgroup of TMA IHC-negative samples, where the whole sections were available from another study (positive ROS1 IHC). Heterogeneity was defined as whole section slides with areas of both 0/1 + in addition to 2+/3+ staining on the whole section or TMA [23].

### Assay methods-NGS

All TMA IHC-positive samples and a subgroup of negative samples were examined with DNA and RNA-sequencing with the next-generation sequencing (NGS) kit Oncomine Comprehensive V3-panel (OCAv3) (Thermo Fisher Scientific, A35806). The negative subgroup consisted of samples with available NGS results from previous/synchronous projects, mainly a study on ROS1 IHC. The library was prepared on an Ion Chef instrument (Thermo Fisher Scientific) and sequenced on the Ion Torrent S5/S5XL system (Thermo Fisher Scientific) at Oslo University Hospital and Vestfold Hospital Trust.

We aimed to use DNA and RNA from fresh frozen tissue stored at  − 80 °C. If the tumor percentage was below 10% and no driver mutation was found (*EGFR-, KRAS- or BRAF*-mutations, or *NTRK-, ROS1-, ALK-* or *RET-*fusions), the sequencing was repeated on DNA/RNA extracted from the original FFPE blocks.

### Assay methods-FISH

We performed FISH on the cases with the highest H-score, except cases with extensive necrosis or autolysis of the tissue in the original FFPE blocks (in total 15 cases). We also included two negative cases for FISH-analysis. The FISH-testing was performed with three different break apart probes; *NTRK* 1–3 Break Apart FISH Probe from Empire Genomics (*NTRK*1BA-20-ORGR, *NTRK*2BA-20-ORGR and *NTRK*3BA-20-ORGR, Empire Genomics, New York, US). FISH was performed according to the protocol from the manufacturer. Fusion-positive tumor was defined with a cutoff of at least 15% split signals, including isolated orange (5`) and green (3`) signals. Fused signals, break apart signals and single green/orange signals were counted in at least 50 nuclei and also in a minimum of 200 signals were counted in each case.

We also wanted to determine if the increased protein expression was due to copy number variation (CNV) not detected with NGS. This was performed with custom gene-specific probes by Empire Genomics (*NTRK*1-20-OR, *NTRK*2-20-OR and *NTRK*3-20-OR, Empire Genomics, New York, US). This FISH-analysis was also performed according to the protocol from the manufacturer.

### Study design and statistical analysis

This is a retrospective cohort study. Associations between epidemiological risk factors, histopathology and IHC-positivity were examined with frequency tables, and univariate and multivariate logistic regression, and two-sided *P* values < 0.05 were regarded as statistically significant. We used Kaplan Meier plots and log-rank tests to assess time to relapse and overall survival. STATA Release 17 [25] was used for statistical analysis.

See the Online Resource for details about the protocols and scoring system.

## Results

**Fig. 1:**
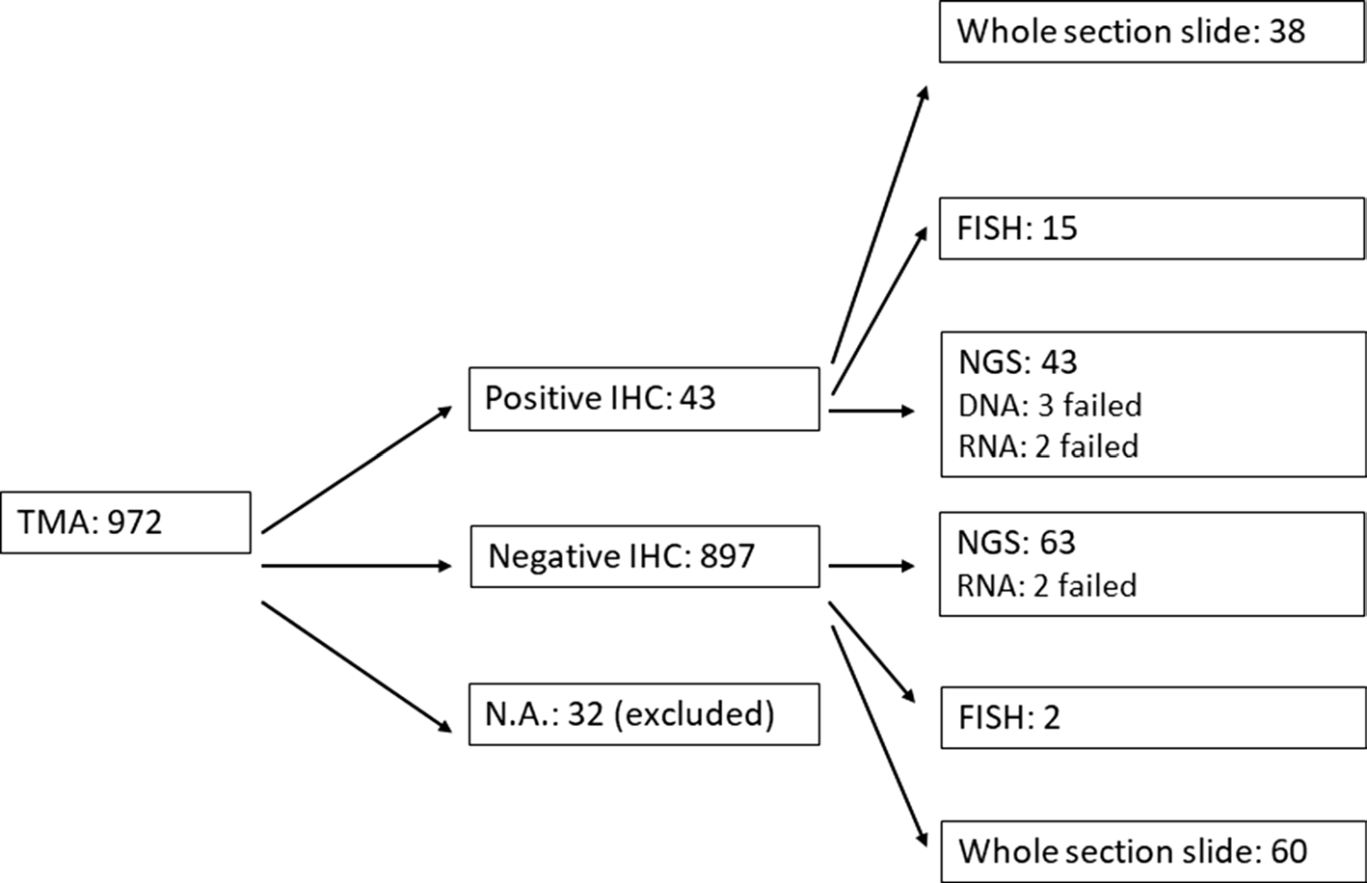
Flowchart. 972 patients were included in the study. Of these 32 samples had too few non-necrotic tumor cells, and were excluded from further analysis. On the 43 IHC-positive cases, we proceeded with NGS and whole section IHC. In a subgroup of the positive samples, we also performed FISH with both break apart probes and CNV probes. We also performed NGS, FISH-and whole section IHC on a subgroup of negative cases

972 patients were included in the study and 36 TMA blocks were made. Samples from 32 patients had too few viable tumor cells in the TMA blocks and were therefore excluded from further analysis (Fig. 1). Of the remaining 940 cases, 477 were men and 463 were women. The median age was 67.5 years. Most patients were current or former smokers (*n* = 859, 91.4%) with a median cumulative pack year of 30.5. The majority were in stages I and II (*n* = 790, 84.0%). Over half of the patients had LUAD (*n* = 523, 55.6%) and about a third had squamous cell carcinoma (LUSC) (*n* = 290, 30.9%) (Table 1).

**Table 1:** Demographic, clinical and histopathological variables at baseline and NTRK IHC

	Total, *n* (%)	Positive IHC	Negative IHC
Total	940 (100%)	43 (100%)	897 (100%)
Age, median years (range)	67.5 (33.9–87.0)	65.2 (43.2–78.4)	67,6 (33.9–87.0)
*Sex*			
Male	477 (50.7%)	30 (69.8%)	447 (49.8%)
Female	463 (49.3%)	13 (30.2%)	450 (50.2%)
*Smoking status*			
Never smoker	81 (8.6%)	2 (4.7%)	79 (8.8%)
Current/former smoker	859 (91.4%)	41 (95.3%)	818 (91.2%)
Median pack year	30.5	38.3	30
*pStage*			
Ia and b	515 (54.8%)	16 (37.2%)	499 (55.6%)
IIa and b	275 (29.3%)	16 (37.2%)	259 (28.9%)
III a and b	138 (14.7%)	11 (25.6%)	127 (14.2%)
IV	11 (1.2%)	0	11 (1.2%)
Unknown stage	1 (0.1%)	0	1 (0.1%)
*Histology*			
Adenocarcinoma (incl. former bronchioalveolar carc.)	523 (55.6%)	6 (14.0%)	517 (57.6%)
Squamous cell carcinoma	290 (30.9%)	30 (69.8%)	260 (29.0%)
Adenosquamous	16 (1.7%)	1 (2.3%)	15 (1.7%)
Carcinoid	49 (5.2%)	2 (4.7%)	47 (5.2%)
Large cell carcinoma	27 (2.8%)	2 (4.7%)	25 (2.8%)
Small cell carcinoma	14 (1.5%)	0	14 (1.6%)
Large cell neuroendocrine carcinoma	7 (0.7%)	0	7 (0.8%)
Salivary gland type carcinoma	5 (0.5%)	2 (4.7%)	3 (0.3%)
Undifferentiated carcinoma	4 (0.4%)	0	4 (0.5%)
Mixed	2 (0.2%)	0	2 (0.2%)
Other	3 (0.3%)	0	3 (0.3%)

Numbers and relative distribution of demographic, clinical and histopathological variables at baseline, stratified by NTRK IHC. The IHC-positive cases were mainly squamous cell carcinoma. 2 of the 5 salivary gland type carcinomas were positive, and these were adenoid cystic carcinomas. Adenocarcinomas were the biggest histopathological subgroup, but only six of these cases were positive

**Fig. 2:**
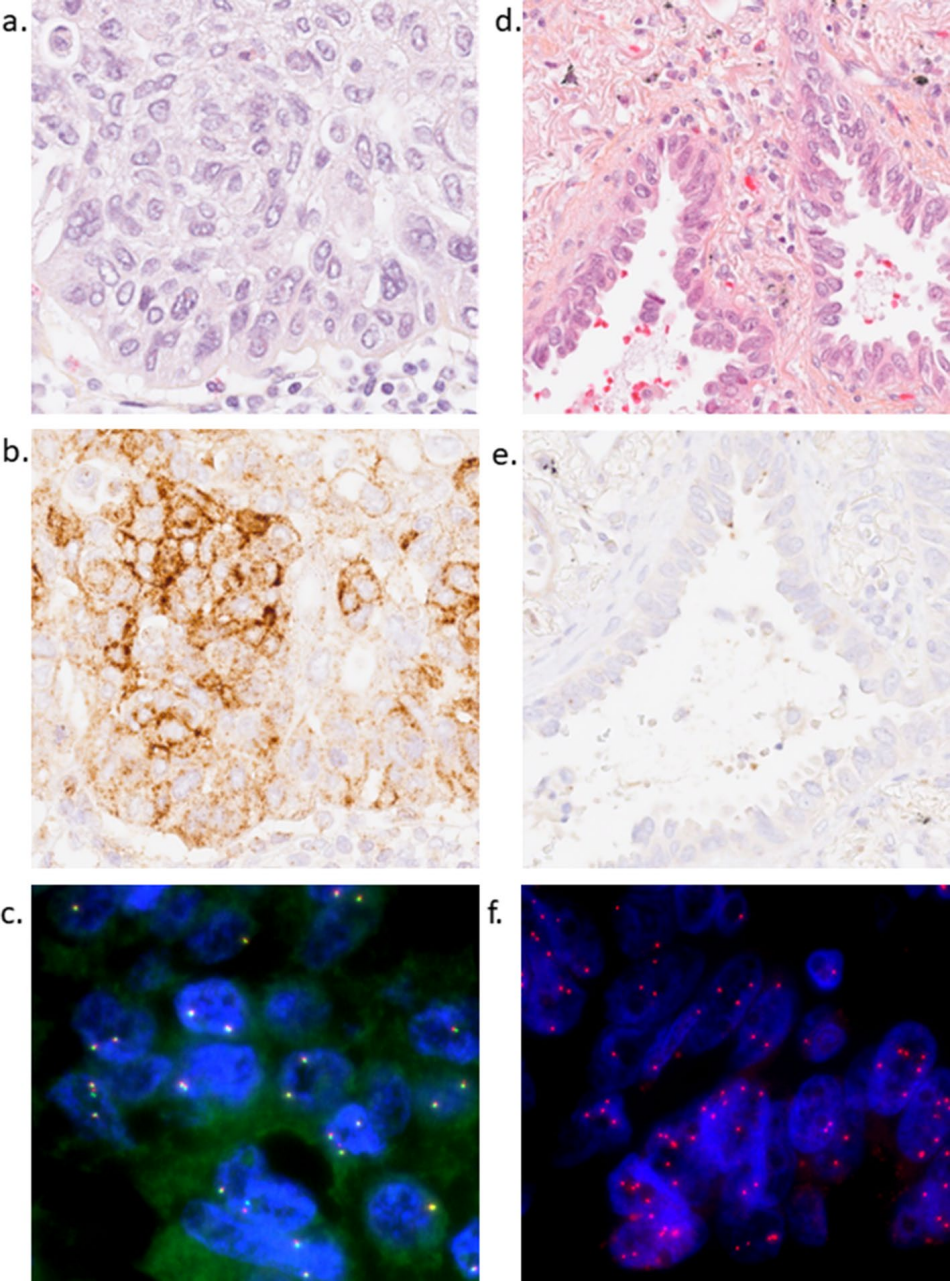
Case with strong and focal IHC and negative FISH (**a-c**). **a** Case with squamous cell carcinoma. Hematoxylin eosin saffron (HES) staining. **b** Same case *NTRK* IHC. Strong, but focal staining. **c** Fused FISH signals, meaning no evidence of NTRK rearrangement/fusion. Case with negative IHC and high copy number with FISH (**d-f**). **d** Case with acinar adenocarcinoma. **e** Same case with negative TRK IHC. Some unspecific staining in macrophages and necrotic cells.** f** *NTRK1* CNV probe. High *NTRK1* copy number (copy number: 8). Picture **a**, **b**, **d** and **e**: 40 × Roche Ventana DP 200 slide scanner

### Immunohistochemistry

43 (4.6%) cases had a positive IHC on the TMAs. Most IHC-positive cases (*n* = 34) had a moderate/strong and focal (median H-score 50) staining, or weak and widespread staining (median H-score 90) (Fig 2). Nine cases had moderate/strong and widespread positive IHC, with a median H-score of 220. The most common staining pattern was cytoplasmatic (evaluated only on cases with available whole sections), but there were also cases with nuclear staining (Tables 1 and 2, and Online Resource Tables 4 and 8).

**Table 2:** Samples with moderate/strong and widespread IHC positivity

	H-score TMA	H-score Whole section	Localization	Histology	Stage	Smoking	Sex
1	300	200	Cytopl	LUSC	Ib	Former	Male
2	190	200	Cytopl	LUSC	Ib	Active	Male
3	190	200	Cytopl	LUSC	Ia	Active	Female
4	230	270	Cytopl	LUAD	Ia	Active	Female
5	280	160	Cytopl	LUSC	IIa	Former	Female
6	220	240	Cytopl	Saliv.g.c	IIa	Former	Female
7	280	300	Cytopl	Saliv.g.c	Ib	Former	Female
8	140	80	Nucleus	LUAD	Ib	Former	Male
9	180	*	*	LUSC	IIa	Active	Male

The cases with strongest positivity on the TMA had mostly a cytoplasmatic staining

LUSC, squamous cell carcinoma; LUAD, lung adenocarcinoma; Saliv.g.c., salivary gland type carcinoma; Cytopl., Cytoplasm

*Whole section slide not available

The interobserver agreement was good, with an observed agreement of 97%. The few discrepant cases were primarily due to different interpretations of non-specific staining in macrophages and necrotic cells.

Of the 43 IHC-positive cases, 30 (69.8%) were LUSC. IHC-positivity was significantly more common in LUSC than in the other histopathological subtypes combined (OR 5.65, 95% CI 2.90–11.01, *p* < 0.001). 30 (69.8%) of the IHC-positive cases were men, but the association between sex and IHC-positivity was non-significant when adjusting for LUSC. Two of the five (40%) salivary gland type carcinomas were IHC-positive, and this association differed significantly from all the non-salivary gland type carcinomas combined (OR 14.54, 95% CI 2.36–89.39, *p* = 0.004). The two positive cases were both adenoid cystic carcinoma, and they showed moderate/strong and widespread, granular and cytoplasmatic staining in 100% of the tumor cells. There were two adenoid cystic carcinomas and one mucoepidermoid carcinoma in the group of IHC-negative salivary gland-type carcinomas. 41 of the patients with positive IHC (95.3%) were current/former smokers, but the association with IHC-positivity did not differ significantly from never smokers (Table 1). 897 cases (95.4%) were defined as IHC-negative. In this group, 19 cases showed focal and weak staining with a median H-score of 20, and 16 of these cases were LUSC.

There were no differences in time to relapse or overall survival between IHC-positive and IHC-negative cases.

Of the 43 TMA IHC-positive cases, original FFPE blocks were available for 38 (88.4%) cases. 30 (78.9%) of these cases were also positive on whole sections, and eight (21.1%) were negative (including weak and focal staining). 24 of the 30 cases that were considered positive on both TMA and whole section, showed heterogeneous staining. Of these 24 cases, 21 were LUSC. In addition, four of the eight cases that were defined as positive in the TMAs and negative in the whole section staining, had areas with 2+/3+ staining in the TMAs, and these were also categorized as heterogeneous. Of the TMA IHC-negative cases, 60 were examined with whole section IHC. Among these, 52 (86.7%) were negative and eight (13.3%) were positive with an H-score between 30 and 170, but none showed moderate/strong and widespread staining. Six of these eight cases were LUSC, one was LUAD and one was salivary gland carcinoma, and all showed heterogeneous staining. In total, 36 (36.7%) cases were considered to show heterogeneous staining, of which 29 (80.6%) were LUSC. The odds for heterogeneity among cases with available whole section slides (*n* = 98) was 28.0 × higher for LUSC compared to non-LUSC (OR = 27.96 CI 9.21–84.87, *p* < 0.001), and the association with LUSC remained when tested on TMA-positive and TMA-negative cases separately.

### DNA and RNA-sequencing

All the defined TMA IHC-positive cases (*n* = 43) were sequenced, two of which failed (Fig. 1 and Table 3). We found no *NTRK* fusions among the IHC-positive cases, nor in the subgroup of sequenced TMA IHC-negative cases (*n* = 63, 2 failed). This group included the eight cases that were negative on the TMA and positive on the whole section. We did find other fusions as described in Table 3. We found numerous different mutations with DNA sequencing, most of which seemed to be randomly distributed (Online Resource Table 6). Four cases with NFE2L2 mutations (R34P, D29H, R34G and G31A) were all TMA IHC-positive and LUSC. We found KRAS mutation in 20 cases. One was LUSC and IHC-positive, and 19 were LUAD and IHC-negative.

**Table 3:** NTRK IHC and results of RNA-sequencing

Scoring group	Totally negative	Focal, weak	Focal, moderate/strong	Widespread, weak	Widespread, moderate/strong
NTRK TMA N (%)	878 (93.4%)	19 (2.0%)	17 (1.8%)	17 (1.8%)	9 (1.0%)
Mean/median H-score (range)	0	24.2/20 (10–40)	58.5/50 (15–120)	94.7/90 (50–150)	223.3/220 (140–300)
RNA NGS number analyzed	62	1	17	17	9
--Fusions	ALK: 1 RET: 1 ROS1: 3 MET ex. 14 skip: 1 Negative: 54 Failed: 2	Negative: 1	FGFR3: 1 Negative: 15 Failed: 1	Negative: 16 Failed: 1	MYB: 1 Negative: 8

No NTRK fusions were found with NGS. The two positive salivary gland carcinomas had strong and widespread positive IHC, and one had a MYB-NFIB fusion and the other a ERBB2 mutation

Met ex 14 skip = Met exon 14 skipping

CNV, copy number variation

### FISH

15 IHC positive cases were analyzed with break apart probes, and no *NTRK*-fusions were found. Most of these cases were LUSC, but also LUAD and salivary gland carcinomas were analyzed. These 15 cases had an H-score between 110 and 300. In addition, two IHC-negative cases were analyzed (LUAD), and no fusion was found in this group either.

With the CNV probes, we found several cases with a high *NTRK*-copy number (Online Resource Table 7). 14 of 17 cases had a high copy number with one or more probes. For NTRK1, 8/17 cases had a high copy number. The highest copy number was 8, and this was in a IHC-negative case. For NTRK 2, 7/17 cases had a high copy number. The highest copy number was 5 and this was found in three IHC positive cases. For NTRK3, 6/17 cases were amplified. The highest copy number was 6, and this was found in three IHC positive cases. There was no association between H-score and high copy numbers. As mentioned, one of the IHC-negative cases had the highest copy number (8, *NTRK*1). *NTRK* amplifications can also be detected by means of NGS, but in this study, no amplifications were found.

## Discussion

In this retrospective study on early-stage lung cancer of various histopathological types, we found no cases with *NTRK*-fusion despite positive immunostaining indicating protein expression in 4.6% of the cases. This suggests that *NTRK* fusion is a rare incident in this cohort. The findings are in line with earlier research. No *NTRK* fusion-positive cases were found in the smaller cohort-studies of surgically resected lung cancer by Elfving et al. and Strohmeier et al. [26, 27]. Other studies have found a prevalence of 0.16–0.23% in NSCLC [12, 17–19], but these studies have been enriched with late-stage cases since previously, genetic testing was usually not conducted in early-stage disease. One might speculate that *NTRK* fusion-positive NSCLC might be more common in late-stage disease and more prone to metastasize. For instance, Farago et al. [17] found 11 cases with *NTRK* fusion in NSCLC, of which eight were in late-stage disease at the time of diagnosis, and only one of the early-stage cases remained recurrence-free. However, our study was not designed nor sufficiently powered to detect any relevant difference in such rare events between early-stage and late-stage lung cancer.

Two of four cases with adenoid cystic carcinoma showed strong and widespread staining. Positive Pan-TRK IHC is common in adenoid cystic carcinoma [12, 28, 29], and this IHC-positivity is not related to *NTRK*-fusion. The positive IHC in adenoid cystic carcinoma might be due to high wildtype TRK-C expression [29, 30]. The salivary gland type tumor subtype secretory carcinoma often has *ETV6-NTRK3*, but this subtype is a very uncommon type of lung cancer [31, 32].

We found a strong association between IHC-positivity and LUSC. The association seems to explain the more frequent IHC-positivity in men. More frequent IHC-positivity in LUSC is consistent with the studies by Elfving et al. and Strohmeier et al. [26, 27]. To the best of our knowledge, the mechanism for this high TRK protein expression in LUSC remains unknown. *NTRK*-fusion is one of many reasons for increased protein expression, and the IHC does not discriminate between wildtype or chimeric (a result of fusion of genes) protein expression. We found no *NTRK* fusions with NGS, and FISH-testing was also negative in the cases with the highest expression. Thus, although the NGS and FISH methods both have limitations, the cases of TRK expression in LUSC in our study seems to be unrelated to *NTRK*-fusion. In lung cancer, *NTRK*-fusion is probably rarer in LUSC than in LUAD. In a cohort study of 4872 NSCLC cases, Farago et al. [17] found only one case of *NTRK* fusion in LUSC compared to 8 cases in LUAD. Similarly, Gatalica et al. found no *NTRK* fusion in LUSC in their cohort study on 4073 NSCLC cases [13]. None of these studies reported the total number of included LUSC. Okamura et al. [16] used data from The Cancer Genome Atlas and found no *NTRK* fusions among 502 cases of LUSC.

Our analysis showed that heterogeneity in staining is very common, particularly in LUSC. Strohmeier et al. and Elfving et al. also found heterogeneity in staining when investigating whole sections in cases without *NTRK* fusion [26, 27]. According to earlier publications, samples with *NTRK* fusion usually show a moderately/strong positivity [12], and at least for *NTRK*1, and the staining is also usually homogenous/widespread [11, 13, 24]. The exception is *ETV6-NTRK3* fusions (mostly found in secretory carcinomas) that can show heterogeneous and often nuclear staining [15], and the result can therefore be falsely negative with IHC [13, 28].

In order to investigate whether CNV could explain the high protein expression with *NTRK* IHC, we used CNV FISH probes and found an increased copy number both in cases with high and with low/no protein expression. Thus, there was no clear association between positive IHC and high copy number. These findings are supported by previous research. Elfving et al. [26] used single-nucleotide polymorphism analysis of gene copy number variations and could not find any cases with amplification (copy number > 4) despite positive protein expression. Solomon et al. [12] found only two IHC-positive cases among 13 cases with *NTRK* CNV and in a study by Lee et al. [33], only four of 27 cases with *NTRK* amplification had a positive pan-TRK IHC.

We found several cases with high copy numbers with the CNV FISH probe, but these were not detected when analyzed with NGS/OCAv3. Detection of CNVs with NGS can be based on several different methods [34, 35]. Ion Reporter Software™ algorithm for detecting CNV relies primarily on the read depth method (RD). The software also requires a baseline workflow and correctly pre-specified tumor percentage. The baseline consists of multiple diverse samples (> 48), in addition to observations in different key variables [36]. The limitation of the RD method arises from insufficient read depth in the target areas. However, given data of sufficient quality, the RD method is highly accurate in detecting both small and large CNVs in all types of regions in a genome [37]. On the other hand, the CNV FISH probe also has its limitations. Since the CNV probe does not include a centromere signal, we cannot exclude that it is polysomy, rather than the amplification of the gene that results in the high copy number. In addition, the probes used also include the neighboring genes, and any amplification of these genes will correspondingly result in high copy numbers [38].

With NGS, we found numerous different mutations, but few patterns. NFE2L2 mutations were exclusively found in IHC-positive cases, whilst KRAS-mutations was most common in the IHC-negative group. This pattern might be explained by the dominance of LUSC in IHC-positive cases and of LUAD in IHC-negative cases. NFE2L2-mutation is known to be more common in LUSC and has been found in approximately 14.0% of LUSC compared with 1.6% of lung LUAD (Datasheet GENIE Cohort v10.0-public) [39–41]. Likewise, KRAS is one of the most commonly mutated genes in LUAD/NSCLC [42].

### Strength and limitations

The most important strength of this study is the large cohort with representative data on surgically resectable lung cancer. Other strengths include performing NGS on all positive cases and the use of FISH on samples with the highest protein expression. The OCAv3 fusion detection is based on RNA-sequencing. It can detect *NTRK*-fusions with a broad range of fusion partners even if the breakpoint is in the intron, and it can potentially also detect non-targeted fusion partners. RNA-quality can be a challenge for fusion detection, but we used primarily fresh frozen material with high RNA-quality. In contrast, the FISH method is DNA-based and can detect fusions irrespective of the fusion partner.

There are several limitations in our study. First, although we scored multiple cores from the same tumor, the screening with IHC on TMAs can potentially miss cases with heterogeneous staining. This is probably most relevant for cases with a *NTRK3-ETV6* fusion [24], but this subtype is uncommon in lung cancer [13, 31]. Secondly, we used a slightly different positive IHC cutoff than in other studies. Several studies have defined positive IHC as staining above background in at least 1% of tumor cells [12, 13, 27]. However, of the 19 cases with focal and weak IHC defined as negative in our study, sixteen were LUSC and therefore less likely to exhibit *NTRK* fusion. Thus, we believe any misclassification bias due to the chosen cutoff level to be negligible. Third, we did whole section IHC, NGS and FISH only on a subgroup of IHC-negative TMA. Although inference might be limited by differences in the distribution of histopathological subtypes (higher prevalence of adenocarcinoma), we believe that the subgroup provides valuable insights into heterogeneity and false negativity. Finally, NGS with OCAv3 can potentially detect fusions with uncommon/non-targeted fusion partners, but there are other methods like FusionPlex by ArcherDX which have shown better sensitivity in this setting [43]. To reduce the probability of false negative results by NGS, we also did FISH-testing on the samples with a high protein expression, and FISH was also negative.

### Conclusion

In this study on early-stage lung cancer, we found several cases with positive IHC indicating TRK protein expression, but no detectable *NTRK* fusions. The study indicates that *NTRK* fusion is rare in early-stage lung cancer. IHC-positivity was not associated with smoking history, time to relapse or overall survival. IHC-positivity was more common in men, but this association seems to be explained by sex differences in LUSC. False positive IHC can show both cytoplasmatic and nuclear staining and is usually heterogeneous. Positive IHC was particularly more common in LUSC and adenoid cystic carcinoma. Pan-TRK IHC is therefore less suited as a screening tool for *NTRK*-fusions in LUSC and adenoid cystic carcinoma.

## Supplementary Information

Below is the link to the electronic supplementary material.

Supplementary file1 (DOCX 43 KB)

## Data Availability

Some data generated or analyzed during this study are included in this published article and supplementary files. Further datasets are not publicly available. However, they can be made available from the corresponding author on reasonable request and after approval by the Regional Ethics Committee.
