# Supplementary material for: Evaluation of NTRK expression and fusions in a large cohort of early-stage lung cancer
Source: Clin Exp Med. 2024 Jan 19;24(1):10. doi: 10.1007/s10238-023-01273-0 (PMC10798916; doi:10.1007/s10238-023-01273-0)
Supplement: Supplementary file 1 — Supplementary file1 (DOCX 43 KB) [file 10238_2023_1273_MOESM1_ESM.docx]

Supplementary information

Article name:

Evaluation of NTRK expression and fusions in a large cohort of early-stage lung cancer

Journal: Clinical and Experimental Medicine

Anne Pernille Harlem Dyrbekk^1,2,3^, Abdirashid Ali Warsame^4^ , Pål Suhrke^2^, Marianne Odnakk Ludahl^5^, Nermin Zecic^5^, Joakim Oliu Moe^6^, Marius Lund-Iversen^4^ and Odd Terje Brustugun^1,3,7^

^1^University of Oslo, NO-0316 Oslo, Norway

^2^Department of Pathology, Vestfold Hospital Trust, NO-3103 Tønsberg, Norway

^3^Department of Cancer Genetics, Institute for Cancer Research, The Norwegian Radium Hospital, NO-0310 Oslo, Norway

^4^Department of Pathology, Oslo University Hospital, The Norwegian Radium Hospital, NO-0310 Oslo, Norway

^5^Department of Microbiology/ Division for gene-technology, Vestfold Hospital Trust, NO-3103 Tønsberg, Norway

^6^Department of Internal medicine, Vestfold Hospital Trust, NO-3103 Tønsberg, Norway

^7^Department of Oncology, Vestre Viken Hospital Trust, NO-3004 Drammen, Norway

Corresponding author:

Anne Pernille Harlem Dyrbekk

Department of Pathology, Vestfold Hospital Trust, NO-3103 Tønsberg, Norway

Email: [anndyr@siv.no](mailto:anndyr@siv.no)

Table of contents

Patient and specimen characteristics, additional information 2

Immunohistochemical protocol 2

Details about the qualitative scoring system 3

Details NGS methods 3

Quality requirements NGS 4

Table 4. Histopathological variables at baseline and NTRK IHC 4

Table 5. Details heterogeneous staining 5

Table 6. Details results DNA NGS 6

Table 7. Details FISH 7

Table 8. Details cases about TMA-IHC staining, including weak and focal. 9

##

## Patient and specimen characteristics, additional information

The study population consisted of patients with surgically resectable lung cancer admitted to surgery at Oslo University Hospital during the period 2006-2018. The population is randomly selected, based on availability of study nurses. The median follow-up time for the patients is exceeding 5 years. Two patients emigrated and were therefore lost to follow up.

Fresh frozen material was obtained after surgery. The samples are approximately 0.5 cc. They were snap frozen in liquid nitrogen and stored in -80°C.

Tissue micro arrays (TMA): The TMAs were made by pathologist at Oslo University Hospital. Core biopsies were obtained from the original FFPE blocks. Each core were one mm in diameter, and it was taken at least three core biopsies from each sample. Each block consists of core biopsies from 25-30 specimens/original FFPE blocks. The TMA blocks were sliced by a special trained Biomedical Laboratory Scientist at the Department of pathology at Oslo University Hospital.

## Immunohistochemical protocol

**Ventana pan-TRK EPR17341 (790-7026)**

| Detection system | Antigen Retrieval | Amplifier | Dilution | Incubation | Staining platform | Counter staining |
| --- | --- | --- | --- | --- | --- | --- |
| OptiView DAB IHC Detection Kit (760-700) | CC1 (On board)  Temp: 100^o^C  88 min | OptiView Amplification Kit (760-099)  4+4 min | Ready to use (RTU) | 32 min  36^o^C | Ventana  Benchmark Ultra | Instant Hematoxilin Kit. Shandon (Ref 6765015)  1 min. Manually. Dilution (1:2) |

## Details about the qualitative scoring system

|  | Group | Definition |
| --- | --- | --- |
| I. | Negative | No visible staining under 400x magnification. |
| II. | Weak (1+) and focal | Light brown staining, visible at 100-200x (10-20x objective), but requiring 400x (40x objective) to see clearly in <50% of tumor cells. |
| III. | Weak (1+) and widespread | Light brown staining, visible at 100-200x (10-20x objective), but requiring 400x (40x objective) to see clearly in >50% of tumor cells. |
| IV. | Moderate/strong (2+ and 3+) and focal | Dark brown staining with 25 - 200x magnification in <50% of tumor cells |
| V. | Moderate/strong (2+ and 3+) and widespread | Dark brown staining with 25 - 200x magnification in >50% of tumor cells |
| VI. | Too few viable tumor cells | <10 viable tumor cells |
| VII. | Ambiguous | Discussed in a consensus meeting |

## Details NGS methods

| DNA and RNA isolation from fresh frozen tissue | AllPrep DNA/RNA/miRNA Universal Kit (Qiagen, 80224) on the QIAcube (automated, spin-column-based nucleic acid extraction from Qiagen) |
| --- | --- |
| DNA and RNA isolation from FFPE blocks | DNA: MagLead12gC (Biosystem, A1120)  RNA: Quick RNA FFPE kit (Zymo Research, R1008) |
| DNA and RNA concentration | Nanodrop (Thermo Fisher Scientific) and Qubit Fluorometer (Thermo Fisher Scientific). |
| RNA quality | Agilent 2100 Bioanalyzer (Agilent Technologies) |
| Ion reporter | Version 5.10-5.16. Filter Oncomine Variants, 5% CI CNV ploidy >= gain of 2 over normal). |

##

## Quality requirements NGS

| **TORRENT SUITE** |  |
| --- | --- |
| % Loading | At least 60% |
| % Polyclonality | 45% or less |
| Low Quality | <20% for DNA, higher for RNA because of degraded material |
| Total Reads | DNA+RNA: 45 000 000-75 000 000 |
| Median reading lenght for DNA | 110-115 bp |
| Medium reading lenght for RNA | 65-110 bp |
|  |  |
| **DNA** |  |
| Mapped Reads | 3 000 000- 6 000 000 |
| % reads on target | >90% |
| Mean depth | >1200 |
| Uniformity | >95%, for degraded material: 85-90% |
| Base coverage depth | >800 bp |
| Number of amplicons | 3781 |
|  |  |
| **RNA** |  |
| Mapped Reads | 500 000-1 500 000 (minimum 40 000) |
|  |  |
| **ION REPORTER** |  |
| **DNA** |  |
| Mutation (QC) | Phred QUAL Score >20  Coverage>1000 |
| CNV | Copy number ≥5 |
|  | MAPD <0,5 |
|  |  |
| **RNA** |  |
| Total Mapped Fusion Panel Reads | >500 000 |
| Expression control counts | Read counts >15 |
| Expression control detected | In total 6 (3 i each pool, should see at least 2-3.) |
| Fusion | Read counts >1000 |
| Skipping and deletions | Read counts >1000 |

## Table 4. Histopathological variables at baseline and NTRK IHC

|  | **Total, n (%)** | **Positive immune** | **Negative immun** |
| --- | --- | --- | --- |
| **Total** | 940 (100%) | 43 (4.6%) | 897 (95.4%) |
| **Histology** |  |  |  |
| Adenocarcinoma (incl. Former bronchioalveolar carc.) | 523 (100%) | 6 (1.1%) | 517 (98.9%) |
| Squamous cell carcinoma | 290 (100%) | 30 (10.3%) | 260 (89.7%) |
| Adenosquamous | 16 (100%) | 1 (6.3%) | 15 (93.8%) |
| Carcinoid | 49 (100%) | 2 (4.1%) | 47 (95.9%) |
| Large cell carcinoma | 27 (100%) | 2 (7.4%) | 25 (93.6%) |
| Small cell carcinoma | 14 (100%) | 0 | 14 (100%) |
| Large cell neuroendocrine carcinoma | 7 (100%) | 0 | 7 (100%) |
| Salivary gland type carcinoma | 5 (100%) | 2 (40.0%) | 3 (60.0%) |
| Undifferentiated carcinoma | 4 (100%) | 0 | 4 (100%) |
| Mixed | 2 (100%) | 0 | 2 (100%) |
| Other | 3 (100%) | 0 | 3 (100%) |

## Table 5. Details heterogeneous staining

|  | TMAs with whole section. Total | Positive TMA and positive whole section | Positive TMA and negative whole section | Negative TMA and positive whole section | Negative TMA and negative whole section |
| --- | --- | --- | --- | --- | --- |
| Total | 98 | 30 | 8 | 8 | 52 |
|  |  |  |  |  |  |
| Heterogeneous cases n (% of total in each column) | 36 (36.7%) | 24 (80%) | 4 (50%) | 8 (100%) | 0 |
|  |  |  |  |  |  |
| Histopathology heterogeneous cases  n (% of total heterogeneous cases) | LUSC: 29 (80.6%)  LUAD: 4 (11.1%)  Ad.sq.c.: 1 (2.8%)  Sal. gl.c.: 1 (2.8%)  Large cell carcinoma: 1 (2.8%) | LUSC: 21 (87.5%)  LUAD: 2 (8.3%)  Ad.sq.c.: 1 (4.2%) | LUSC: 2 (50.0%)  LUAD: 1 (25.0%)  Large cell carcinoma: 1 (25.0%) | LUSC: 6 (75.0%)  Sal. gl.c.:: 1 (12.5%)  LUAD: 1 (12.5%) |  |
| Non-heterogeneous cases n (% of total in each column) | 62 (63.3%) | 6 (20%) | 4 (50%) | 0 | 52 (100%) |
|  |  |  |  |  |  |
| Histopathology non-heterogeneous cases  N (% of total non-heterogeneous cases) | LUAD: 49 (79.0%)  LUSC: 8 (12.9%)  Ad.sq.c.: 0  Sal. gl.c.: 4 (6.5%)  Large cell carcinoma: 1 (1.6%) | LUAD:2 (33.3%)  Sal. gl.c.: 2 (33.3%)  LUSC: 2 (33.3%) | LUSC: 2 (50.0%)  LUAD: 1 (25.0%)  Large cell carcinoma: 1 (25.0%) |  | LUAD: 46 (88.5%)  Sal. gl.c.: 2 (3.8%)  LUSC:: 4 (7.7%) |

The distribution of heterogeneity in cases with whole section material. LUSC: Squamous cell carcinoma Ad.sq.c.: Adenosquamous carcinoma Sal. gl.c.: Salivary gland type carcinoma LUAD: Lung adenocarcinoma

## Table 6. Details results DNA NGS

| Scoring group | Totally negative | Focal, weak | Focal, moderate/strong | Widespread, weak | Widespread, moderate/strong |
| --- | --- | --- | --- | --- | --- |
| DNA NGS  Number analyzed | 62 | 1 | 17 | 17 | 9 |
| --Mutations | CREBBP: 1  RB1: 4  TP53: 26  PTEN: 1  RAD50: 2  SETD2: 3  ARID1A: 2  EGFR: 8  BRCA2: 1  ERBB2: 1  FBXW7: 1  IDH1: 1  NOTCH1: 1  STK11: 2  KRAS: 19  PIK3R1: 1  PTCH1: 3  ATRX: 1  CDK4: 1  STAT3: 1  TSC2: 1  TSC1: 1  Negative: 9 | TP53: 1 | TP53: 9  NFE2L2: 2  PTEN:2  SETD2: 1  ARID1A: 2  EGFR: 2  ESR1: 1  STK11: 1  FANCD2: 1  KRAS: 1  PIK3CA: 3  PIK3R1: 1  PTCH1: 1  CDKN2A: 1  SLX4: 1  Negative: 2  Failed: 1 | TP53: 7  NFE2L2: 2  PTEN: 1  RAD50: 2  SETD2: 2  EGFR: 1  FBXW7: 1  PTCH1: 1  Negative: 5  Failed: 2 | CREBBP:1  MET: 1  RB1: 1  TP53: 3  ERBB2: 1  NOTCH1: 1  Negative: 4 |
| --CNV | FGFR1: 1  AKT2: 1  AKT3: 1  CDK6: 1  MDM2: 1  MYC: 1  MYCL: 1  PIK3CA: 1  TERT: 1  EGFR: 1  KIT: 1  PDGFRA: 2  RICTOR: 1  Negative: 53 | Negative: 1 | Negative: 16  Failed: 1 | FGFR1: 1  Negative: 13  Failed: 3 | CCND1: 1  CCNE1: 1  FGFR3: 1  AKT2: 1  FGF19: 1  Negative: 7 |

Met ex 14 skip = Met exon 14 skipping

CNV: copy number variation

## Table 7. Details FISH

| Nr | **NTRK1**  H-score whole section slide | Histopath. | DNA NGS | RNA NGS | Copynumbr. (copies/nucl.) | **~** | Split signals | Nuclei | %  fused signals | %  split signal | Conclusion |
| --- | --- | --- | --- | --- | --- | --- | --- | --- | --- | --- | --- |
| 1 | 300 | Saliv.g.c | Neg | NF1B-MYB fusion | 200/68 = 2,94 | 3 | 200 | 98 | 0,93 | 0,07 | Amplified |
| 2 | 270 | LUAD | RB1 mut. | Neg | 200/101 = 1,98 | 2 | 200 | 82 | 0,98 | 0,01 | Not amplified |
| 3 | 240 | Saliv.g.c | TP53, ERBB2 mut., CCBE1 and AKT2 CNV | Neg | 200/96 = 2,08 | 2 | 200 | 94 | 0,93 | 0,07 | Not amplified |
| 4 | 200 | LUSC | Neg | Neg | 200/36 = 5,55 | 6 | 200 | 38 | 0,95 | 0,05 | Amplified |
| 5 | 200 | LUSC | TP53 mut. | Neg | 200/28 = 7,14 | 7 | 200 | 48 | 0,97 | 0,03 | Amplified |
| 6 | 190 | LUSC | ARID1A and CDKN2A mut. | Neg | 200/98 = 2,04 | 2 | 200 | 111 | 0,98 | 0,02 | Not amplified |
| 7 | 160 | LUSC | Neg | Neg | 200/60 = 3,33 | 3 | 200 | 58 | 0,98 | 0,02 | Amplified |
| 8 | 150 | LUSC | Neg | Neg | 200/119 = 1,68 | 2 | 200 | 108 | 0,96 | 0,04 | Not amplified |
| 9 | 170 | LUSC | PTEN mut. | Neg | 200/48 = 4.17 | 4 | 200 | 52 | 0,93 | 0,07 | Amplified |
| 10 | 140 | LUSC | PIK3CA and TP53 mut. | Neg | 200/48 = 4,17 | 4 | 200 | 106 | 0,94 | 0,06 | Amplified |
| 11 | 130 | LUSC | TP53 mut. | Neg | 200/154 =1,30 | 1 | 200 | 126 | 0,96 | 0,04 | Not amplified |
| 12 | 140 | LUSC | ESR1, TP53 and STK11 mut. | Neg | 200/114 = 1,75 | 2 | 200 | 106 | 0,94 | 0,04 | Not amplified |
| 13 | 140 | LUSC | TP53 mut. | Neg | 200/102 = 1,96 | 2 | 200 | 103 | 0,95 | 0,05 | Not amplified |
| 14 | 110 | LUSC | SETD2 mut. | Neg | 200/95 = 2,10 | 2 | 200 | 106 | 0,92 | 0,08 | Not amplified |
| 15 | 110 | LUSC | TP53 mut. | Neg | 200/98 =2,04 | 2 | 200 | 108 | 0,96 | 0,04 | Not amplified |
| 16 | 0 | LUAD | KRAS mut. | Neg | 200/26 =7,69 | 8 | 200 | 61 | 0,98 | 0,02 | Amplified |
| 17 | 0 | LUAD | KRAS mut. | Neg | 200/48 = 4,16 | 4 | 200 | 60 | 0,97 | 0,03 | Amplified |

| **NR** | **NTRK 2**  H-score full size slide | Copynumber (copies/nucleis) | Round number  **~** | Split signals | Nuclei | Percentage fused signals | Percentage split signal | Conclusion |
| --- | --- | --- | --- | --- | --- | --- | --- | --- |
| 1 | 300 | 200/102 = 2,22 | 2 | 200 | 90 | 0,99 | 0,01 | Not amplified |
| 2 | 270 | 200/51 = 3,92 | 4 | 200 | 106 | 0,96 | 0,04 | Amplified |
| 3 | 240 | 200/92 = 2,17 | 2 | 200 | 98 | 0,98 | 0,02 | Not amplified |
| 4 | 200 | 200/42 = 4,76 | 5 | 200 | 46 | 0,97 | 0,03 | Amplified |
| 5 | 200 | 200/38 = 5,26 | 5 | 200 | 78 | 0,98 | 0,02 | Amplified |
| 6 | 190 | 200/108 = 1,85 | 2 | 200 | 80 | 0,99 | 0,01 | Not amplified |
| 7 | 160 | 200/58 = 3,455 | 4 | 200 | 98 | 0,97 | 0,03 | Amplified |
| 8 | 150 | 200/98 =2,04 | 2 | 200 | 112 | 0,97 | 0,03 | Not amplified |
| 9 | 170 | 200/52 = 3,35 | 3 | 200 | 98 | 0,98 | 0,02 | Amplified |
| 10 | 140 | 200/92 = 2,17 | 2 | 200 | 92 | 0,96 | 0,04 | Not amplified |
| 11 | 130 | 200/83 = 2,41 | 2 | 200 | 86 | 0,98 | 0,02 | Not amplified |
| 12 | 140 | 200/40 = 5 | 5 | 200 | 61 | 0,99 | 0,01 | Amplified |
| 13 | 140 | 200/52 = 3,84 | 4 | 200 | 75 | 0,98 | 0,02 | Amplified |
| 14 | 110 | 200/102 = 1,98 | 2 | 200 | 90 | 0,99 | 0,01 | Not amplified |
| 15 | 110 | 200/72 = 2,78 | 3 | 200 | 94 | 0,98 | 0,02 | Amplified |
| 16 | 0 | 200/96 = 2,1 | 2 | 200 | 104 | 0,99 | 0,01 | Not amplified |
| 17 | 0 | 200/124 = 1,60 | 2 | 200 | 132 | 0,97 | 0,03 | Not amplified |

| **NR** | **NTRK3**  H-score full size slide | Copynumber (copies/nuclei) | Round number  **~** | Split signals | Nuclei | Percentage fused signals | Percentage split signal | Conclusion |
| --- | --- | --- | --- | --- | --- | --- | --- | --- |
| 1 | 300 | 200/98 = 2,04 | 2 | 200 | 70 | 0,99 | 0,01 | Not amplified |
| 2 | 270 | 200/42 = 4,76 | 5 | 200 | 102 | 0,97 | 0,03 | Amplified |
| 3 | 240 | 200/96 = 2,08 | 2 | 200 | 103 | 0,99 | 0,01 | Not amplified |
| 4 | 200 | 200/32 = 6,25 | 6 | 200 | 61 | 0,98 | 0,02 | Amplified |
| 5 | 200 | 200/32 = 6,25 | 6 | 200 | 50 | 0,99 | 0,01 | Amplified |
| 6 | 190 | 200/36 = 5,555 | 6 | 200 | 46 | 0,98 | 0,02 | Amplified |
| 7 | 160 | 200/100 = 2 | 2 | 200 | 95 | 0,99 | 0,01 | Not amplified |
| 8 | 150 | 200/104 = 1,92 | 2 | 200 | 106 | 0,99 | 0,01 | Not amplified |
| 9 | 170 | 200/104 = 1,92 | 2 | 200 | 110 | 0,97 | 0,03 | Not amplified |
| 10 | 140 | 200/88 = 2,27 | 2 | 200 | 87 | 0,95 | 0,05 | Not amplified |
| 11 | 130 | 200/106=1,89 | 2 | 200 | 88 | 0,97 | 0,03 | Not amplified |
| 12 | 140 | 200/43 = 4,65 | 5 | 200 | 58 | 0,98 | 0,02 | Amplified |
| 13 | 140 | 200/98 = 2,04 | 2 | 200 | 74 | 0,98 | 0,02 | Not amplified |
| 14 | 110 | 200/39 = 5,13 | 5 | 200 | 64 | 0,98 | 0,02 | Amplified |
| 15 | 110 | 200/86 = 2,32 | 2 | 200 | 112 | 0,99 | 0,01 | Not amplified |
| 16 | 0 | 200/102 = 1,96 | 2 | 200 | 107 | 0,98 | 0,02 | Not amplified |
| 17 | 0 | 200/143 = 1,39 | 1 | 200 | 98 | 0,97 | 0,03 | Not amplified |

##

## Table 8. Details cases about TMA-IHC staining, including weak and focal.

| **Moderate/strong and widespread** | | | | | | |
| --- | --- | --- | --- | --- | --- | --- |
| Nr | H-score TMA | H-score Full size | Histology | Stage | Smoking | Sex |
| 1 | 300 | 200 | LUSC | Ib | Former | Male |
| 2 | 190 | 200 | LUSC | Ib | Active | Male |
| 3 | 190 | 200 | LUSC | Ia | Active | Female |
| 4 | 230 | 270 | LUAD | Ia | Active | Female |
| 5 | 280 | 160 | LUSC | IIa | Former | Female |
| 6 | 220 | 240 | Saliv.g.c. | IIa | Former | Female |
| 7 | 280 | 300 | Saliv.g.c. | Ib | Former | Female |
| 8 | 140 | 80 | LUAD | Ib | Former | Male |
| 9 | 180 | - | LUSC | IIa | Active | Male |
|  |  |  |  |  |  |  |

| **Weak and widespread** | | | | | | | |
| --- | --- | --- | --- | --- | --- | --- | --- |
| nr | H-score TMA | H-score Full size | Localization | Histology | Stage | Smoking | Sex |
| 10 | 70 | 0 | - | LUSC | IIIa | Former | Male |
| 11 | 50 | 225 | Cytopl. | LUSC | IIa | Former | female |
| 12 | 80 | 100 | Cytopl. | LUSC | IIIa | Former | Male |
| 13 | 100 | 110 | Cytopl. | LUSC | Ia | Current | Male |
| 14 | 80 | 0 | - | LUAD | Ia | Former | female |
| 15 | 80 | 0 | - | LUSC | IIIa | Former | Male |
| 16 | 100 | 30 | Cytopl. | LUSC | Ia | Former | Male |
| 17 | 100 | 120 | Cytopl. | Adenosquamous | IIa | Current | female |
| 18 | 130 | 90 | Cytopl. | LUSC | Ib | Former | Male |
| 19 | 150 | 130 | Cytopl. | LUSC | IIb | Current | Male |
| 20 | 120 | 70 | Cytopl. | LUSC | IIIa | Current | Male |
| 21 | 100 | 150 | Cytopl. | LUSC | IIa | Former | female |
| 22 | 90 | 90 | Cytopl. | LUSC | IIb | Current | Male |
| 23 | 80 | 0 | - | LUAD | IIIa | Current | Male |
| 24 | 90 | - | - | Carcinoid | IIa | Never | Male |
| 25 | 90 | 0 | - | Large cell carcinoma | IIa | Former | Male |
| 26 | 100 | - | - | LUSC | IIb | Former | Male |
|  |  |  |  |  |  |  |  |

| **Moderate/strong and focal** | | | | | | | |
| --- | --- | --- | --- | --- | --- | --- | --- |
| Nr | H-score TMA | H-score Full size | Localization | Histology | Stage | Smoking | Sex |
|  |  |  |  |  |  |  |  |
| 27 | 40 | 0 | Cytopl. and nucleus | Large cell carcinoma | Ib | Current | Male |
| 28 | 70 | 2 | Cytopl. | LUAD | IIIa | Never | Female |
| 29 | 110 | 30 | Cytopl. | LUSC | IIb | Former | Male |
| 30 | 100 | 40 | Cytopl. and nucleus | LUSC | IIIa | Former | Male |
| 31 | 40 | 10 | Nucleus | LUSC | IIIa | Former | Male |
| 32 | 15 | 5 | Cytopl. | LUSC | Ib | Current | Female |
| 33 | 55 | 0 | Cytopl. | LUAD | IIb | Former | Female |
| 34 | 30 | 30 | Cytopl. | LUSC | Ib | Current | Male |
| 35 | 30 | 5 | Cytopl. | LUSC | Ia | Current | Male |
| 36 | 40 | 20 | Cytopl. | LUSC | Ib | Current | Male |
| 37 | 35 | 30 | Nucleus | LUSC | IIa | Current | Male |
| 38 | 60 | 0 | Cytopl. | LUSC | IIb | Current | Male |
| 39 | 50 | 10 |  | LUSC | IIIa | Current | Male |
| 40 | 40 | - |  | Carcinoid | IIIa | Former | Male |
| 41 | 120 | - | Cytopl. | LUSC | IIIa | Current | Male |
| 42 | 100 | 10 | Cytopl. | LUSC | Ib | Former | Female |
| 43 | 60 | 50 | Cytopl. | LUSC | IIa | Current | Male |

| **Weak and focal** | | | | | | | |
| --- | --- | --- | --- | --- | --- | --- | --- |
| Nr | H-score TMA | H-score Full size | Localization | Histology | Stage | Smoking | Sex |
| 44 | 20 |  |  | LUSC | IIIa | Former | Female |
| 45 | 30 |  |  | Carcinoid | Ia | Former | Male |
| 46 | 40 |  |  | LUSC | Ib | Never | Male |
| 47 | 20 |  |  | LUSC | IIa | Current | Male |
| 48 | 20 | 30 | Cytopl. | LUSC | Ib | Current | Male |
| 49 | 20 |  |  | LUSC | IIa | Current | Male |
| 50 | 10 |  |  | LUSC | IIa | Current | Male |
| 51 | 30 |  |  | LUSC | Ia | Former | Male |
| 52 | 20 |  |  | LUSC | IIIa | Current | Male |
| 53 | 29 | 140 | Cytopl, | LUSC | Ia | Current | Male |
| 54 | 30 |  |  | LUSC | Ib | Former | Male |
| 55 | 20 |  |  | LUSC | Ia | Current | Male |
| 56 | 30 |  |  | LUAD | IIb | Current | Male |
| 57 | 30 |  |  | LUSC | Ib | Current | Male |
| 58 | 40 |  |  | LUSC | IIa | Former | Male |
| 59 | 30 |  |  | LUSC | IIIa | Former | Female |
| 60 | 10 |  |  | LUSC | Ia | Former | Male |
| 61 | 20 |  |  | LUSC | Ia | Former | Male |
| 62 | 10 |  |  | Carcinoid | Ia | Never | Male |
